# Socioeconomic Inequalities in Informal Care Provision and Its Consequences for Well-Being Across Europe

**DOI:** 10.1093/geroni/igaa057.2567

**Published:** 2020-12-16

**Authors:** Nekehia Quashie, Judith Kaschowitz, Christian Deindl, Martina Brandt

**Affiliations:** 1 TU Dortmund, Dortmund, Germany; 2 TU Dortmund, Dortmund, Nordrhein-Westfalen, Germany; 3 Heinrich-Heine-Universität Düsseldorf, Duesseldorf, Nordrhein-Westfalen, Germany

## Abstract

We assess socioeconomic inequalities in informal care provision and its consequences for the wellbeing of informal caregivers. The literature states that a lower socio economic status (SES) is linked to a higher probability to give care (at higher intensities) which then leads to a higher caregiving burden. People with lower SES additionally have fewer resources to alleviate caregiving pressures. Thus, they are likely to experience decreased wellbeing compared to those with higher SES. Our analyses based on data from SHARE and ELSA confirm, that individuals with lower SES are indeed more likely to provide care all over Europe. They also report a lower wellbeing than people with higher SES, even if controlling for further important influences. In the next step we investigate longitudinally, if taking over care responsibilities leads to a wellbeing decline and if this decline is more pronounced for people with lower SES.

